# The International Exhibition

**Published:** 1862-07

**Authors:** 


### Art. II.—THE INTERNATIONAL EXHIBITION.

When these pages see the light, the great event of the year will have passed through two months of its ephemeral existence, and extensive material whereby to form a correct judgment of the history of the past decade, and to indulge in an augury of the future, will have been collected by every reflective and analytic mind. It is not our purpose to dwell upon those more salient features of the second holding of the World's Fair, which must arrest the attention and claim the admiration of all comers, which are commented upon by individuals and by coteries, which illustrate the design of the founders, and force themselves upon the least cultivated perceptions. It is enough to render our passing tribute to the public spirit, and the intellectual acumen which actuated and invigorated the designers of this great scheme: it suffices to say, with the rest of the world, "Well done;" and then we may pass to those considerations which most naturally suggest themselves to the mind that regards the International Exhibition of 1862 less as a fact than an influence, less as a great scheme successfully carried to its issue, than as an important link in a chain of association, as a lasting landmark in the history of mankind.

With the purpose and effect of the International Exhibition in a commercial point of view, it is not our province to deal, while we fully acknowledge the grandeur of the scale upon which its arrangements have been effected, the immense national and international importance of the scheme, and the wide results which it is calculated to develope. In a certain sense, the commercial and the educational value of such an undertaking are identical; and it is in this sense that we propose to examine its purpose and results.

The design of this great work, like most mighty conceptions, is equally vast and simple. It proposes to represent the world, as it is now, in the sixth millennary of its existence; to present an illustrative tableau of the civilization of the globe at this date. As such, it is an educational institution of the highest importance and the utmost value, enclosing within its limits all that books can teach only in outline, and placing their contents within the estimation of the least cultivated, while offering them, with all their associations, to the observation of those who are best versed in what may be called the *dead languages* of such studies. In this form they live. The student of human cultivation and development has but to wander in spirit from one part of the habitable globe to another, throughout its extent, and he may find the representative specimens of progress in their most characteristic and instructive forms. Under this roof, inscribed with its fitting tribute of ascription to the Almighty Creator of all things, the student finds the types of races and civilizations, of manners, customs, progress, wealth, social relations, and intellectual development, from the finished luxury and refinement of the Western Empires, to the subtle but barbaric splendour of the Oriental climes, and the first faint assimilation by the Polynesian nations of the rudiments of that social science which has reached its height in these favoured countries. There is the vast yet simple beauty of completeness in this grand design, which has borrowed the truth and utility of industry, whereon to rear the superstructure of the beauty and the elevating majesty of Art. Its scope is at once catholic and eclectic, for while it omits no source from whence comment on its great meaning may be drawn, in each it has chosen the highest, the most finished, and the best. In these long and spacious aisles, the artisan finds the articles which his hands have fashioned, and the tools with which he has worked, in their most perfect condition of completeness; he can estimate in their entirety and value the fabrics which he has only hitherto seen under the coarsest aspect; he can realize the meaning and the dignity of the system of which he forms a portion; he can gauge the industrial resources of his own country, and compare them with those of other lands; if not accurately, or with much technical skill, at least with sufficient expansion of mind to convey to him an added interest in, a more extensive comprehension of, his daily task. At least his eyes are strengthened and refreshed by the sight of the gay and beautiful combinations before him: there is a certain refinement inseparable from such perceptions, which must blend with the external toil and monotony of the artisan's life. It is, perhaps, more curious and interesting to speculate upon the effect which such a scene must produce upon the mind of the unlearned visitor, than to contemplate the sources of enjoyment which it has to offer to the educated. To the latter it is only a comment—an eloquent, majestic, sublime comment, it is true; only geography, history, political economy, commerce, poetry, and art, with numberless illustrations. To the unlearned man, to the gazer who brings to the contemplation of the scene merely the rudimentary knowledge of certain facts for which alone a life of toil supplies opportunity, it is a revelation. The artisan has heard of foreign countries, but he knows them vaguely by name, perhaps chiefly by some piece of technical information connected with his handicraft, at best slightly, and with none of the realizing aid of travel or literature. When he visits the International Exhibition, he begins to understand them. He is a cabinet-maker, perhaps, and France is realised to him by her buhl and marqueterie, her room-hangings, and decorations. He examines them curiously, marks the various woods, observes the designs, compares them with the models from which he works; is curious about the tools; can see them, too, can compare them with those he uses, and perceive the differences and the varieties; and justly estimate the condition of his own particular trade in each country—for here he sees the best in every department. Nor is his observation confined to the representative specimens in his own branch of industry. It is not only the cabinet-making of France that he sees, but every fabric which that productive and ingenious country can show: he cannot fail, the first appeal to his perceptive faculties having been made through the unfailing agency of the familiar, to wander delightedly among the strange, even mysterious objects surrounding him. Can that British workman ever again belong to the class which associates the idea of France with frogs and "parley-voo?" Even a short visit to such a scene, with all its entirely novel impressions, though doubtless bewildering, must also be enlightening, must give form and substance to mere vague notions and outlines. The mind of the uneducated man is seldom attuned to the perception of beauty; it requires a link of association to connect it with the objects offered to its perception, and that link must be forged by custom. The things he sees and handles daily in their rudest and plainest, are presented to him in this emporium of manufactures, in their highest, most refined, and most beautiful forms. The educational process to which nations are subjected is identical with that which each individual mind must undergo: it is a system of stages and degrees, a thing of comparisons and competitions, now slow and anon rapid; and in this illustrative scene the workings of both are brought under our observation. The impression to be made on the uneducated mind by the scene which the International Exhibition presents is essentially, in its beginnings, an educational one; moral and political considerations come after. The working man's life is necessarily monotonous, and his sphere of observation is compulsorily narrow. Here is an opportunity to offer to his perception at once a number of objects, and a succession of ideas, far greater and more striking than could ever be conveyed by books, supposing his leisure permitted or his taste indicated their perusal; and far more valuable in their results than any reading could be, because their appeal is directed to those faculties which are most developed by the conditions of his life. To a certain extent, he is at home among the objects which surround him; beyond that limit their strangeness is not confusing, though full of beauty. He has the clue to the labyrinth of lavish colour, of graceful form, of exquisite convenience, of elaborate finish; he is to some extent in the secret of this brilliance and luxury. Then his mind begins its journey into the kingdom of knowledge, passing the boundary of his handicraft, and reaching forward to the study of the work of other men's hands. He may not go far; but the impetus will have been given, the perceptions enlarged, the spirit of inquiry awakened, that peculiar process of intellectual advance begun, which is not again to be arrested, though it may be slackened for want of opportunity, and rendered slow by the pressure of toil.

The moral effect of such a scene upon the uneducated man is a most interesting subject of contemplation. He does not come to its inspection with certain set notions and class opinions, founded on previous observation and anticipative of certain results. The moral bearing, the ethical meaning, of the vast design which he contemplates has no distinct existence for him. As it slowly unfolds its details to his perceptions, its significance grows upon him in greater sublimity because he has not a plan of its moral measurement in his mind. He acquires an idea of the design of the world, the meaning of life, the inexorable law of human industry and endeavour, the wide-spread sources of gain, and resources of enterprise; the community of human interests, the indissoluble links of human society, and the appointed place of each man in the working of the great machine whose Maker and Artificer is God. He sees in the crowded phantasmagoria moving round him, all sorts and conditions of men; he hears the sound of strange languages, and sees the reflex of his own sentiments of admiration, curiosity, inquiry, or surprise, in the foreign faces. He feels, he has it forced upon his perception, that the tie of brotherhood which unites these children of Our Father is not a bond of nationality or tongue, but of wider extent and deeper significance. The great cosmopolitan gathering of All Nations should be mighty to strike at the root of prejudice and narrowness, to let in a full strong gleam of the light which shall shine to the perfect day. When the uneducated man inspects the beautiful, the precious, the useful objects of industry in the International Exhibition, it is not with a morose and jealous sense of the difference between the rich and the poor, and a hostile perception of the luxury of life in the one class and its privations in the other. The scheme is too thorough, its educational method has been too deeply studied, too skilfully carried out, to render such a result probable, in any cases of even fairly average intelligence. The working man sees there, by indisputable illustration, that the same progress, the same industry, the same liberality of enterprise, the same freedom of institutions and enlightened spirit of trade which have invented and perfected every conceivable luxury for the mansions of the rich, have brought comfort, and even refinement, into the dwellings of the poor. The mysterious ordination of Providence still divides the world, with many subdivisions, into these two classes—the rich and the poor; and such division must remain, as its Ruler has told us: but the progress of society and the advance of knowledge and industry have elevated each in their degree. The splendid decorations, the profuse articles of personal luxury, the gorgeous appanage of rank and wealth, with which the Courts of the International Exhibition are crowded, are not more striking, and are, except in their artistic aspect, even less interesting than the numberless means and appliances of simple household comfort by means of which a higher state of civilization is grafted upon the conditions of toil and poverty. The cottage of to-day may contain, in its simple and scanty furniture, refining forms of grace and elegance unknown to the manor-house when the dwellers in each were children. Besides, the workman knows that the increase of luxury to the rich implies the increase of comfort to the poor. In all these beautiful and brilliant things he sees the handiwork of himself and his fellows—he feels his place and his importance. The form is indeed gigantic and of excessive height and beauty, but he and such as he are its thews and sinews. As he stands under the vast roof of the Palace of Industry, the working man is in his rightful place.

The political effect of the International Exhibition on the minds of the working classes of England, who, even when uneducated, in the technical sense of the term, are notoriously alive to political events, and keen to receive political impressions, is calculated to be of the most useful and the happiest kind. The Exhibition is a matter-of-fact proof of the political blessings and benefits of the Government under which the English nation lives; and it is just such a proof as the sensible and straightforward English nation can thoroughly appreciate and perfectly comprehend. It is a fact which no demagogue can gainsay—a solid actuality which men can see and touch, and which, while it is justly flattering to their pride, is a candid and unanswerable appeal to their common sense. It is a response to the query, "How far does the British system of government meet the requirements of political science—the greatest good of the greatest number?" It is a friendly challenge to the other nations of the civilized world, that they should come and compare the extent to which arts, sciences, industry, and the refinements and adornments of life have progressed among them, with the point to which we have reached. It is a candid interchange of lessons and suggestions, a philosophical *séance* for the examination of human progress on the grandest scale. Surely, the English nation has reason to sustain the ordeal with equal confidence in its rulers and in itself. It is not in a vainglorious spirit that we consider the great national and political privileges to which the International Exhibition bears its majestic witness. England is pre-eminently the land of solid comforts and of well-ordered and respectable homes. Other lands have finer dwellings, costlier and more beautiful fabrics to show; but we can challenge the world to exhibit the same amount of refinement and comfort placed within the reach of the lower and working classes. It is in his own national department that the English working man will especially feel at home; while from the foreign departments he will acquire a new perception of beauty, and a larger comprehension of those conditions of life beyond his personal view.

Passing from this branch of our subject, on which we have said only enough to suggest a few not unproductive topics of thought and themes of investigation, it may not be uninteresting to study the International Exhibition in the aspect under which it presents itself to the educated mind. Mechanical aids to study must always be highly appreciated by those whose desire for knowledge is too keen and constant to permit them to remain satisfied with the vague, the abstract, or the general. The prayer of Ajax finds reiteration in all purely intellectual aspirations. To know, to have light, to see with one's mental and bodily eyes the things of which books tell us, is the most earnest and legitimate longing of the cultivated mind. The same sentiment which has so frequently animated the religious, does not fail to find life and strength in the literary or artistic enthusiast. It finds its most ordinary and frequent expression in travel, and has been so recognised as to become proverbial. "Vedi Napoli, e poi morir," says the amour propre of one country; "Quien non ha vista Seviglia, non ha vista Maraviglia," says the sententious self-satisfaction of another. The natural expression of education and of awakened taste is towards the expansion and the realization to be attained by travel. Travel is the fanaticism of education, as it was the fanaticism of religion, in the days when the good knights whose "bones are dust," whose "swords are rust," aspired to that simplest, yet grandest of epitaphs, "Saw Hierosalem." Thus we, too, holding, in books, the keys of the citadels of knowledge, would fain explore them by travel. We have a company of great names, which are but shadowy companions of our intellectual wayfaring, and the eye and ear experiences of travel turn them into living things. History is no longer ghostly; its paths lie through a valley of dry bones no more, when the student reads it, as he follows in the triumphal track of the Roman legions, or in that of the modern civilization which has beautified, but not displaced, the majestic ruins of the old world. The past and present live together in the mind of the traveller who carries with him to the realization of all that has hitherto been familiar only by the medium of books, strong powers of observation, and those faculties of association which are among the most pleasure-giving of our intellectual gifts. But this deepening and finishing touch to the fabric of education is within the reach of comparatively few. Even in this locomotive age, the proportion of educated persons, with keen and cultivated tastes, and strong desire for knowledge, who are unable to procure the advantages and delights of travel, to those within whose reach they are placed, is small. It would be difficult to imagine any expedient by which the privation inflicted by the absence of such facilities could be so far obviated, as by that of the International Exhibition. It combines several of the peculiarly intellectual advantages to be gained by the personal study of foreign countries, in a philosophical point of view, as many persons might not combine them by an unassisted mental and imaginative effort. The social meaning, the historical, political, and commercial status of each country is to be read here, in their representative industrial products and artistic achievements. Physical features are indeed wanting; but, after all, these are precisely what it is most possible to realize from books, with the assistance of painting; but the mind, the brain, the vitality of each country are here laid bare to every observer familiar with the mere outline of its characteristics. The amount of assistance afforded by such an emphatic comment upon abstract lessons, can scarcely be estimated even by the learner. His mind absorbs the easy, though great knowledge offered to it, without effort; for it is so presented by all the avenues of the senses, that it exacts no toil or endeavour. It is a just, though it may seem an ignoble, comparison, to liken the International Exhibition to the modern system of maps; for it is in a similar way, though in an incomparably greater degree, that it teaches. As in a modern map, the geological strata, then the physical features; thirdly, the political and national boundaries; fourthly, the traffic system; and lastly, the large buildings and important localities are marked out, so that the student may become gradually familiar with the places he is investigating,—this great industrial and commercial tableau is progressive and all-embracing. Here each country is represented by a process of development which, when steadily followed up, leaves a sense of knowledge, familiarity, and completeness, which, superseding that which is gained by reading, must, in the future, lend to books an additional and fascinating interest. Henceforth, to close students of the lessons of the International Exhibition, books which tell of the life and meaning, the progress and the arts of other countries than his own, will be no longer *genre* pictures, to be curiously scrutinized, but portraits to be recognised and criticised.

To the mind which loves to study men more than things, there is an area of observation, a field of thought, within these decorated walls, rich in curious problems and interesting speculations. Here are the transcripts of men's minds, the men of every country, of every political, intellectual, and educational condition. Here is to be read their ambition, their enterprise, the bent of their tastes, the genius of their industry, the animating motive of their endeavours. Here, amid all that is new, graceful, and of modern application in invention, in manufacture, in the trappings and adornments of life, may yet be traced the deathless, if dim, traditional features of each race and nation. It does not need the embroidered banner and foreign names to tell the visitor to the French Court whither his steps have led him; the gay and graceful, the ingenious, decoration-loving, gloom-abhorring spirit has set its sign upon every inch of the space allotted, as legibly as in the spacious, lightsome, gilded galleries of the palaces which witnessed so much that was fine and frivolous in France. Is not the love of state and show, the devotion to externals, and the exquisite sense of the suitable and the beautiful in all things, which are inseparable from the French national character, to be read in everything there? Nor is a view of the reverse of the medal wanting. Let the observer scan the treasures of literary enterprise which the French nation has contributed, and he will be as much struck with the elaborate completeness of its work, with the stamp of "thorough" which is on all it undertakes and accomplishes. Amid the numerous signs of the wonderful commercial and industrious revival which has been accomplished by the political resuscitation of Spain, there is no difficulty in tracing the proud and graceful sombreness, the reticent dignity of the old Spanish character. While its fabrics attest the rapid change of the commercial aspects of the country, and its furniture and decorations testify to its adaptation to the modern taste of other lands, a glance at its art-contributions carries the visitor back to the old days when the Spanish hidalgo was the stateliest, gloomiest, proudest, and most reserved of men. Perhaps the artists of Spain do not seek their subjects from history and tradition more exclusively than those of other nations, but they are narrower in their choice. The artistic mind seems to run in one particular groove, and to be tinged as deeply now as in the days of Velasquez with the grave, sombre tints of loyalty to misfortune, and strength in suffering and death. And Italy! Italy in her renewed youth, and the fresh glory of her nationality, what of her who seems to have broken the spell of the "dono infelice di tanta bellezza"? She sends beautiful things, means and appliances of modern luxury, and rare handiwork of cunning craftsmen; but the old classical spirit, the noble patrician touch is upon them; the love and pride of tradition is in every shape and form. A walk through the Italian Courts is most truly instructive and illustrative, when taken in the spirit in which Nathaniel Hawthorne writes of Italy, and with a cherished remembrance of the days before great Pan was dead. To this Department many great and various interests attach themselves. While it manifests the mind of the present with force and vividness which the peculiar circumstances of Italian politics lend to its representation, it is loftily reminiscent of the past.

To the *habitué*, the poetical and speculative aspect of the beautiful and diversified scene will doubtless soon become familiar. In it he will freely indulge, finding each day and hour new food for contemplation of his fellow men, and feeling more and more deeply the harmonizing and liberalizing influence of the great display of common interests, and of individual efforts directed to general results. It may be safely predicated, that all the ranks and conditions into which the human family is divided will be represented there, except, perhaps, those which have fallen, through vice or suffering, into an isolation from which even this great reunion cannot snatch them, for a little, into the fellowship of their kind. And what is the International Exhibition more forcibly, than an appeal for such as these? "Men and brethren" is the title, sacred in pagan antiquity, clarion-tongued in the first fierce fight of Christianity against the world, the watchword of freedom, and the impulse of progress now, by which this great triumph, warning, protestation, and appeal, solicit widened sympathies and quickened comprehension for the ignorance, the sin, the degradation, and the wretchedness lurking and skulking among us, never to learn the lessons this palace of philosophy and social science has to teach, unless we transmit them.

While they are, in one sense, unconnected with the moral and actual meaning of the International Exhibition, the Art-treasures, which present such unfailing attractions to the throngs of visitors, are, in another, deeply significant and interesting. Apart from their beauty, their testimony to the extraordinary progress of the age in Art, they have the significance of their position as completing the educational process of this design. In them, the observer who has studied all the manifestations of the different countries represented here, reaches their highest and noblest evidence. When he has sought into and studied the minds of his fellows in all that scientific invention, machinery, manufactures, and every branch of industry, can show, he has but to raise his eyes to the wealth-laden walls of the picture-galleries, or to let them rest on the long lines of statuary which lend their chaste and imposing beauty to the scene, and he reaches the standard of each country's genius and imagination as well. Here, too, he may study at once the sources and the results of the inspiration of the artistic mind; here he may read national characteristics, as well as individual tastes, and, with many a backward glance through the "corridors of time," trace the gradual purification and elevation of Art into the expression of truth, liberty, and brotherhood. It would be idle to speak of the magnitude and importance of the collection of Art-treasures, useless to allude to that which has been dwelt upon with unanimous astonishment and delight since the opening of the International Exhibition. They present a spectacle no more possible to describe than to forget.

There is another portion of the inexhaustible subject of which we have been able to offer but a poor, pale outline, meagre in design and weak in execution, but perhaps possessing some little power of suggestion, which it is impossible to pass over in silence, and difficult to mention aright. A great writer, when he had dwelt long upon the merits of a great painter, learned that he had just died. He added to his eloquent exposition and panegyric, words which would have formed a fitting epitaph to be placed upon the tomb of Joseph Mallord William Turner, in which he prophesied that the famous year of 1851 should be "remembered less for what it had given than for what it had taken away." Eleven years have passed, and the same may be said with tenfold significance of 1862. The opening of the International Exhibition, amid all its pomp and splendour, through its music and its glitter, through its rejoicing and its grandeur, was less a festival than a commemorative solemnity. Its deepest meaning lay in its deepest deprivation; and it may be said, in the words of Bossuet: "Et rien enfin ne manque, dans tous ces honneurs, que celui à qui on les rend."

---

